# Post-ERCP acute pancreatitis and its risk factors

**Published:** 2013-03-25

**Authors:** A Iorgulescu, I Sandu, F Turcu, N Iordache

**Affiliations:** General Surgery Clinic, "Sf. Ioan" Hospital, Bucharest

**Keywords:** ERCP, post ERCP complications, post ERCP acute pancreatitis, risk factors

## Abstract

Introduction. Endoscopic retrograde cholangiopancreatography (ERCP) is a complex endoscopic technique that evolved from a diagnostic to a mainly therapeutic procedure. This was due to the identification of post-procedural complications that can follow both simple ERCP and that associated with the instrumentation of the biliary and pancreatic ductals. The identification of post ERCP complications in a proportion of 5 to 10% of cases, with a mortality rate of 0.33%, imposed their analysis and study of risk factors involved in their occurrence. The significance of post ERCP complications reveals the necessity of their avoidance by adopting additional measures if risk factors are identified.

Materials and methods. We have retrospectively analyzed 900 cases that underwent ERCP in the Surgery Department of "Sf. Ioan" Clinical Hospital in a period of 17 years. The complications of the procedure were studied. Among them, a special attention was given to post-ERCP acute pancreatitis (pERCP-AP), the most common complication that occurred in the study group. We also tried to find out and highlight the risk factors for this complication.

Results. ERCP is a relatively safe invasive procedure, yet it has complications (8% of cases), some of them potentially fatal (mortality 0.43%). The most common complications after ERCP are acute pancreatitis (3.7%), papillary bleeding (1.04%), retroperitoneal duodenal perforation (0.69%) and biliary septic complications like acute cholecystitis and cholangitis (1.21%). Acute pancreatitis is by far the most common complication. Risk factors for its occurrence are difficult sphincterotomy with precut use, failure of CBD desobstruction, pancreatic sphincterotomy, repeated injection of contrast in the pancreatic ductal system, dysfunction of the sphincter of Oddi and the absence of changes of chronic pancreatitis. When risk factors are identified, the patients’ selection must be very strict and diagnostic ERCP should be avoided in favor of non-invasive diagnostic methods (MRI-cholangiography, echo-endoscopy).

## Introduction

ERCP has become an almost exclusively therapeutic endoscopic procedure. Of all the gastrointestinal endoscopic interventional techniques, ERCP recorded the highest rate of complications. Reported data show great variability. This is because the different authors do not use a unitary definition of these complications, the methods of collection and processing of data are diverse and so are the procedure’s indications and technique. 

 Multiple studies [**1-3**] report post-ERCP complications’ incidence for specific complications (acute pancreatitis, papillary bleeding, retroperitoneal duodenal perforation and biliary septic complications like acute cholecystitis and cholangitis) and non-specific ones.

 A review published by Andriulli [**1**] in 2007, analyzing 21 studies that enrolled 16855 patients, for a period of 7 years, gives 6.9% rate of specific complications, which lead to a death rate of 0.33%. The same author brings together 14 studies with 12973 patients, in which the percentage of non-specific complications is 1.3 and the mortality rate is 0.07%. The data are relevant to the importance and severity of complications of ERCP. The most common of these is pERCPAP.

 The incidence of such complications varies even in prospective studies. In 1998, Loperfido [**4**] reports a rate of 0.74% for pERCPAP after diagnostic procedures and 1.4% after therapeutic ones. More recent studies published by Wang [**5**] in 2009, mention an incidence of 4.3%, summing up both the diagnostic and therapeutic procedures.

 In the consensus classification published in 1991 by Cotton et al. [**6**] PApERCP is defined as the clinical syndrome suggestive to acute pancreatitis (intense abdominal pain newly developed or exacerbated) accompanied by amylasemia over 3 times the normal range for at least 24 hours and requesting more than an overnight hospitalization.

**Table 1:** Consensus Classification of Post-ERCP Acute Pancreatitis

	Mild	Moderate	Severe
Post-ERCP Acute Pancreatitis	• Clinical picture of pancreatitis • Persistent (over 24h) amylasemia – over 3 times the normal range • Requires hospitalization for at least 2-3 days	• Requires hospitalization for 4-10 days	• Requires admission in ICU • Hospitalization for more than 10 days • Pseudocyst • The need for invasive treatment (percutaneous drainage or open surgery)

The definition is succinct, but does not allow the diagnosis of all the patients, ignoring those who, although they have severe abdominal pain, do not have an amylasemia that is high enough, as well as those asymptomatic patients with very high values of serum amylases.

 The pathophysiological mechanisms involved in the productalion of pERCP-AP are multiple and their degree of involvement is variable. They can be summarized as it follows:

 1. mechanical obstruction of the pancreatic drainage through: 

a. mechanical injury of the pancreatic sphincter or proximal main pancreatic ductal 

 b. pancreatic sphincter edema caused by sphincterotomy or thermal injury

 c. prolonged sphincter spasm in patients with sphincter hypertonia. 

 2. Hydrostatic hyper-pressure in pancreatic ductal system: 

 a. excessive injection of contrast

 b. pancreatic manometry without distal aspiration

 c. extrinsic compression of the main pancreatic ductal through a distal choledochal obstructive calculus

 3. pancreatic bacterial contamination by: 

 a. duodenal contents

 b. lack of compliance with antiseptic measures during the procedure

Once triggered by one of these mechanisms, the process of extra ductal enzymatic activation will result in the occurrence of acute pancreatitis. 

 The degree of involvement of one or other of the mechanisms is unknown, therefore repeated attempts were undertaken to identify the independent factors involved in the occurrence of this complication.

 Two categories of factors were considered: some patient related and other method related.

 Patient-related factors that proved to be involved in the genesis of pERCP-AP in several multi-centre clinical trials [**2,4-11**] are the following:

 • Sphincter of Oddi dysfunction

 • Young age

 • Normal values of bilirubinemia

 • History of pERCP-AP.

 Sphincter of Oddi dysfunction is the condition most often revealed as a risk factor for pERCP-AP [**2-11**]. It triples the risk of such complication and acts as an independent factor. Data published by Freeman in 2001 show that the risk of complication in patients who have sphincter of Oddi dysfunction is the same regardless of whether the procedure is performed for diagnostic or therapeutic purposes. 

 Women seem to have a higher risk of developing pERCP-AP [**10-11**], but not all the studies have proven this. The contribution of sphincter of Oddi dysfunction was taken into consideration, this being present almost exclusively in the female sex. The association of these risk factors is cumulative and it additionally increases the risk of developing pERCP-AP.

 Normal values of bilirubinemia represent a risk factor [**11**]. The absence of stones in patients with suspected choledochal lithiasis also seems to be a risk factor. The explanation could be represented by the erroneous interpretation of the etiology. Sphincter of Oddi dysfunction can generate symptoms and may actually represent the predisposing condition to the appearance of complication.

 History of pERCP-AP is also an important risk factor with Odd Ratio (OR) between 2 and 3.4 on multicentric studies [**7,10,11**]. Absence of chronic pancreatitis, though not very solidly statistically proven, can be a risk factor. The explanation can be the pancreatic tissue atrophy and reduced enzymatic secretion present in this pathologic entity.

 Age less than 60 years predispose to the appearance of pERCP-AP. A possible explanation lies in increased enzymatic activity at young.

Factors related to the technique of ERCP have been studied for long in order to prove their involvement in the appearance of specific complications. The risk of complications is similar for diagnostic and therapeutic procedures [2,4,7-11]; hence, the recommendation to avoid diagnostic ERCP is fully justified, as long as there are non-invasive diagnostic methods for most of these illnesses.

 Papillary trauma by repeated cannulation attempts during difficult interventions is an independent risk factor for the occurrence of pERCP-AP. Its effect is cumulative with that of ductal hyper-pressure induced by repeated injections of contrast [**2-11**]. 

 An inflammatory process, whose evolution can be ameliorated by providing decompression of the pancreatic ductal tree [**7-11**], often follows pancreatic sphincter trauma.

 The use of precut technique represents a risk factor for pERCP-AP [**2-4**]. A limited number of endoscopists (10-30%) practice it. The use of this approach in difficult cases, in which repeated cannulation attempts failed, may be an explanation for the statistical results. A randomized trial conducted in a tertiary unit showed that there are no significant differences between the rates of complications of repeated attempts of cannulation and the use of precocious precut technique [**13**]. On the other hand, Freeman demonstrates that increased risk associated with precut sphincterotomy is operator dependent [**12**].

 Presented data show very clearly the need for prevention of pERCP-AP. The most important and reliable way to avoid complications is by restraining from using ERCP of those patients who do not have firm indications and especially of those who have risk factors. 

## Materials and methods

We retrospectively analyzed 900 cases in which ERCP was performed in the Department of General Surgery of "Sf. Ioan" Clinical Hospital Bucharest from 1st of June 1994 to 31st of December 2009. Of these, 75 patients have had acute biliary pancreatitis as an indication for ERCP and were eliminated from the study.

 The work was based on an analysis of the patients' observation sheets, as well as on the analysis of the operation registry. We analyzed thepatients’ demographics, indication of the procedure, the peculiarities of intervention and post-operative evolution, paying special attention to post-ERCP complications. We aimed to identify the risk factors that could be implied in pERCP-AP and their degree of involvement. We used Odd Ratio (OR) and confidence interval (95% CI) to assess the degree of implication of the different risk factors.

## Results and discussion

The work includes 1,150 patients admitted in the Department of General Surgery of "Sf. Ioan" Clinical Hospital Bucharest between 1st of June 1994 and 1st of January 2012, in whom ERCP was performed. We analyzed the patients’ demographics. The average age was 62.3 years, with limits between 14 and 88 years. The sex distribution rate was of 55.2% in women and of 44.8% in men.

 The spectrum of diagnoses for which the procedure was indicated is shown in Table 2

**Table 2:** Indications of ERCP

Diagnosis	% of cases	Comments
Biliary acute pancreatitis	6.5%	75 patients who were excluded from the study
Choledochal lithiasis	68%	
Postoperative biliary fistula	8.3%	
Benign or malignant choledochal stenosis	12.1%	
Sphincter of Oddi stenosis	5.1%	

**Fig. 1:**
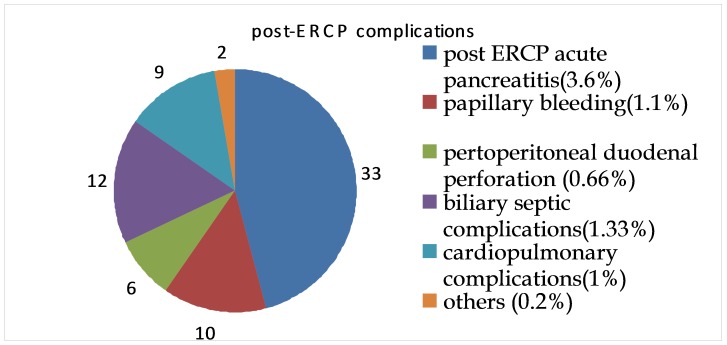
Post-ERCP complications

 In the studied group, the incidence of post-ERCP complications was of 8%. The different complications are shown in **Fig. 1**.

The mortality rate in the study group was of 0.5% (5 cases), concordant with the data reported in literature. Death was caused by the above-mentioned complications.

**Table 3:** Post ERCP deaths

Diagnosis	Indication of ERCP	Nr. of cases
pERCP-AP	Benign stenosis of distal choledochus	1
Septic complications (cholangitis)	Malignant stenosis	2
Cardio-pulmonary complications	CBD lithiasis with cardiopulmonary comorbidities	2

We have registered a case of pERCP-AP in a patient with interposed duodenal diverticulum, with distal choledochal stenosis, CBD dilatation and jaundice. Repeated cannulation attempts failed and the procedure was abandoned, but the papillary trauma triggered a very severe form of necrotic pancreatitis with multiple organ failure and exitus despite sustained treatment.

 Septic complications - cholangitis - occurred after endoscopic stenting of two malignant high choledochal stenoses that, despite the establishment of an efficient biliary drainage and sustained antibiotherapy, evolved to severe sepsis and death.

 Cardiopulmonary complications were recorded in fragile patients in whom the endoscopic maneuver cured choledochal lithiasis, but the decompensation of their comorbidities could not be avoided or treated.

 The mortality rate must be analyzed in the context of the pathology to which ERCP addresses, in comparison with mortality in the absence of the maneuver and with that of therapeutic alternatives.

 pERCP-AP was the most common complication, having an incidence of 2.3%. The data obtained are consistent with those in literature, where the incidence varies between 1% and 7% [**2,4,6,9,11**].

 We considered the diagnosis of pERCP-AP in situations in which the abdominal pain appeared or worsened, associated with an increase of amylasemia over 3 times the upper limit of normal range (3 x 110U/l), within the first 24 hours post ERCP and necessitated a hospitalization of minimum 48 hours. The majority of the cases have been mild forms of pancreatitis with remission under conservative treatment: fasting and nasogastric drainage, hydric, electrolytic and energetic support, antisecretory treatment and antimicrobial prophylaxis. We have registered a case of acute necrotic pancreatitis mentioned in the deaths.

 We evaluated the following risk factors implied in the occurrence of pERCP-AP:

 • difficult sphincterotomy (precut usage)

 • failure of choledochal clearing

 • pancreatic sphincterotomy

 • repeated injection of contrast into the pancreatic ductal system

 • sphincter of Oddi dysfunction

 • female sex

 • absence of chronic pancreatitis

 We used Odd Ratio (OR) and the confidence interval (95% CI) to assess the degree of involvement of risk factors in the occurrence of pERCP-AP. The results are presented in Table 4:

**Table 4:** Risk factors for the occurrence of pERCP-AP

Risk factors	Adjusted OR	95% CI
Difficult sphincterotomy (precut usage)	3.48	2.55 - 4.75
Failure of choledochal clearing	3.2	1.47 - 6.93
Pancreatic sphincterotomy	2.98	1.5 - 3.5
≥ 1 injection of contrast into the pancreatic ductalal system	2.28	1.14 - 4.56
Sphincter of Oddi dysfunction	2.64	1.23 - 5.67
Female sex	1.1	1.4 - 2.05
Absence of chronic pancreatitis	1.3	1.17 - 1.51

The most important risk factors are those related to endoscopic technique. Excessive papillary trauma in the absence of its cannulation, with precut papillotomy (to locate the choledochal pore), has proven to be an important risk factor for pERCP-AP, having an OR of 3.84 and a CI of 2.55-4.75. A notable proportion of these patients had sphincter of Oddi dysfunction, which is an independent risk factor for pERCP-AP (OR 2.64) and the most important patient-related risk factor.

Returning to the endoscopic technique, pancreatic sphincter trauma, even if it makes a pancreatic sphincterotomy, as well as repeated injection of contrast into the pancreatic ductals, increase the risk of postoperative pancreatic inflammation.

That is why some authors [**7-11**] recommend Wirsung ductal stenting if the procedure results in repeated trauma or pancreatic sphincterotomy. This maneuver does not eliminate the risk of post ERCP pancreatitis, but mild forms will be developed. However, pERCP-AP occurs in 2.5% of cases even after maneuvers in which no contrast had been injected into the pancreatic ductals [**11**].

Another significant risk factor is failure of choledochal clearing (OR 3.2 and 95% CI 1.47-6.93). If choledochal clearing failed, choledochal stenting may be a temporary solution, also having a protective role against pERCP-AP.

The absence of chronic pancreatitis and female gender are moderately involved as risk factors for pERCP-AP, with OR 1.3 and, respectively, 1.7. Freeman considers that chronic pancreatitis is a protective factor against post ERCP pancreatitis, his arguments being the parenchymal atrophy and reduced enzymatic secretion present in the illness [**11**]. On the other hand, the same author, as well as Cheng [**7-11**], consider the history of pERCP-AP a potential risk factor, with an OR of 2.0-5.4.

The incidence and spectrum of post ERCP complications and, among these, pERCP-AP with its different forms, require serious consideration of the maneuver’s indications. Referral to this procedure only for therapeutic maneuvers, after clearly establishing the diagnosis by non-invasive methods, represents a fully justified precaution.

Being aware of risk factors and pointing them out routinely enable us to avoid marginal indications of ERCP and/or add some supplementary preventive measures against complications.

## Conclusion

ERCP is a relatively safe, invasive procedure, but it has risks of complications (8%), some of which are potentially fatal (mortality rate of 0.43%).

The most common post ERCP complications are acute pancreatitis (most frequently encountered, 46.7% of total), hemorrhage, perforation and biliary septic complications (cholangitis and acute cholecystitis).

 Some of the risk factors for pERCP-AP can be preoperatively identified: female sex, sphincter of Oddi dysfunction, absence of chronic pancreatitis. In these circumstances, the selection of patients must be very strict and diagnostic ERCPs should be avoided, favoring non-invasive diagnostic methods (cholangio - MRI, endoscopic ultrasonography).
